# Genome Sequences of Microviruses Identified in a Sample from a Sewage Treatment Oxidation Pond

**DOI:** 10.1128/MRA.00373-21

**Published:** 2021-05-13

**Authors:** Simona Kraberger, Joshua Schreck, Craig Galilee, Arvind Varsani

**Affiliations:** a The Biodesign Center for Fundamental and Applied Microbiomics, Center for Evolution and Medicine, School of Life Sciences, Arizona State University, Tempe, Arizona, USA; b School of Biological Sciences, University of Canterbury, Christchurch, New Zealand; c Structural Biology Research Unit, Department of Integrative Biomedical Sciences, University of Cape Town, Cape Town, South Africa; Queens College CUNY

## Abstract

Oxidation ponds are often used in the treatment of sewage as an aeration step prior to discharge. We identified 99 microvirus genomes from a sample from a sewage oxidation pond. This diverse group of microviruses expands our knowledge of bacteriophages associated with sewage oxidation pond ecosystems.

## ANNOUNCEMENT

Treated and untreated sewage or wastewater contains a multitude of microorganisms that are excreted from humans and the environment. Given the rich bacterial communities present in sewage, viruses that infect bacteria, called bacteriophages, are common (1, 2). Bacteriophages play an important role in the treatment process (3–6) by modulating bacterial populations. They have also been used as an indicator of fecal pollution (7, 8). Many studies have also identified bacteriophages from sewage as potential therapeutics against antibiotic-resistant bacteria (9, 10).

**FIG 1:**
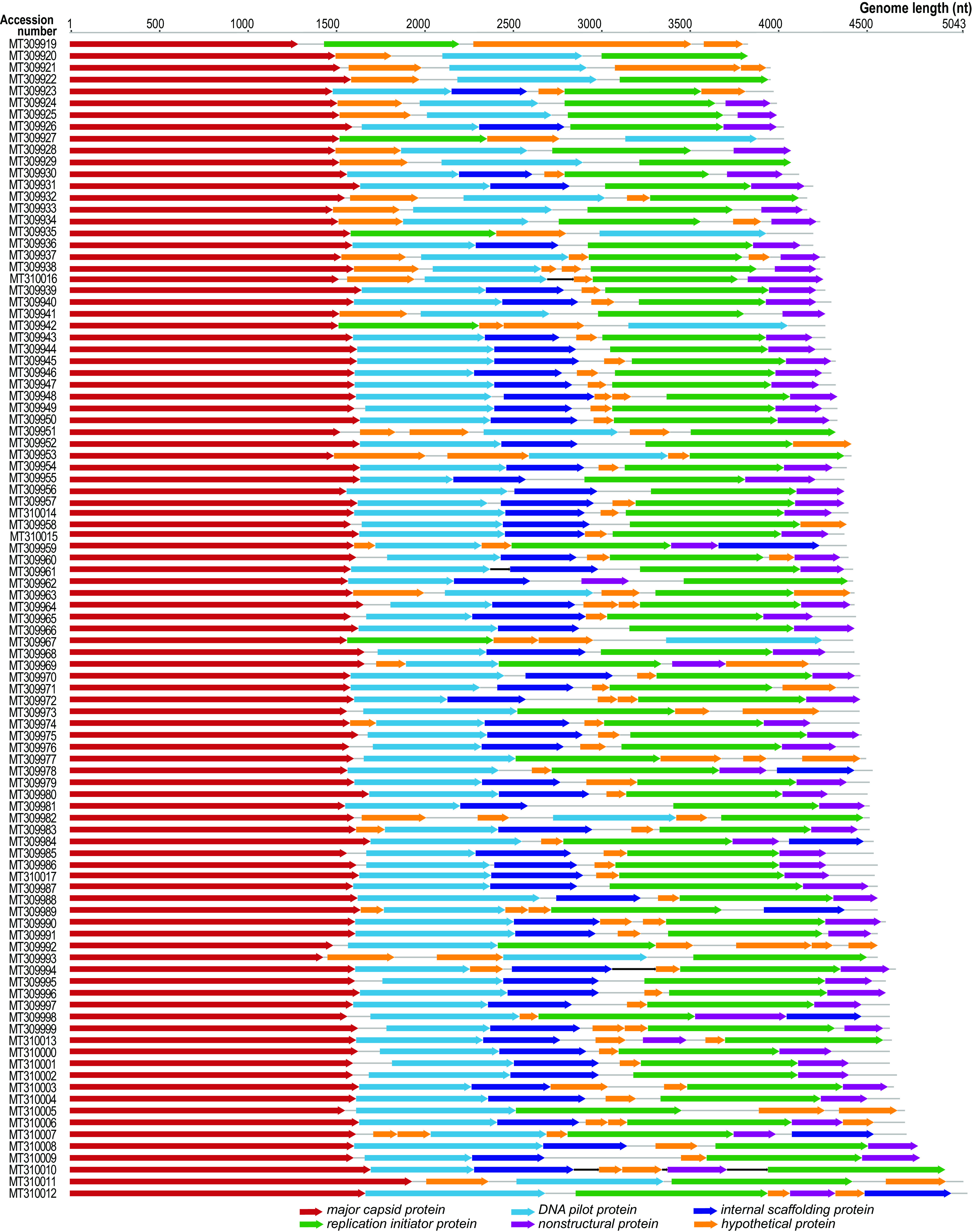
Genome organization (linear depiction) of the 99 microviruses identified in this study.

In September 2012, a 50-ml sample was collected from an oxidation pond at the Christchurch wastewater treatment plant in New Zealand (Aotearoa), transported on ice, and processed. Nucleic acids were isolated as described by Kraberger et al. (11). A total 50 ml of the sample was sequentially filtered through a 0.45-μm and 0.2-μm syringe filter, and the filtrate was precipitated with 15% (wt/vol) polyethylene glycol at 4°C overnight. The precipitate was pelleted by centrifugation at 10,000 × *g* for 10 min and then resuspended in 1 ml of SM buffer (0.1 M NaCl and 50 mM Tris-HCl [pH 7.4]), and 200 μl of this suspension was used to extract viral DNA using the High Pure viral nucleic acid kit (Roche Diagnostics, USA). The extracted circular viral DNA was preferentially amplified by rolling circle amplification (RCR) with the TempliPhi 100 kit (GE Healthcare, USA). The RCR products were sequenced at Beijing Genomics Institute (Hong Kong, China) on the Illumina HiSeq 2000 platform using the propriety library preparation workflow with an insert size of 170 bp with 91-bp paired-end reads. Raw reads (26,757,090) were quality trimmed with Trimmomatic v 0.39 (12) and *de novo* assembled using metaSPAdes v 3.12.0 (13). The eukaryotic viruses, mainly those in the phylum *Cressdnaviricota* (14), from this sample have been previously described by Kraberger et al. (11). To determine the prokaryotic viruses in this sample, contigs were analyzed using VirSorter 2.2.1. (15). Contigs with terminal redundancy were deemed complete circular genomes, and reads were mapped back to full genomes using BBMap (16) for verification and to determine the number of mapped reads and depth of coverage. Default parameters were used for all software unless otherwise specified. Ninety-nine full unique microvirus genomes were identified, with an average genome coverage ranging from 9- to 2,879-fold (Fig. 1).

*Microviridae* is a family of singled-stranded DNA viruses with circular genomes (17) that have been identified in a broad range of samples types and environments (18–31), including sewage (3, 32, 33). Two subfamilies have been established in the family, namely, *Bullavirinae* and *Gokushovirinae*. In this study, we aimed to expand our knowledge on microviruses in sewage by specifically looking at those in the secondary stages of sewage treatment where it is aerated in an oxidation pond prior to being dispersed into the environment.

The microvirus genomes range in length from 3,797 to 5,043 nucleotides (nt), with a GC content of ∼33% to 60% (Table 1). Open reading frames were determined using RASTtk (34), with annotation being determined using BLASTp (35) similarities with GenBank-curated microvirus RefSeq proteins. All genomes encode at least a major capsid protein (MCP) and a replication initiator protein (Fig. 1). Additional proteins were also identified in some of the genomes, which include DNA pilot proteins, nonstructural proteins, and internal scaffolding proteins. A data set of the MCP sequences from the microviruses in this study and those available in GenBank (17 February 2021) was assembled. Analysis of this data set revealed that major capsid proteins from this study share 36.9% to 98.4% pairwise amino acid identity, as determined using BLASTp (35), with all others in the data set (Table 1).

**TABLE 1:** Organization summary of the 99 microvirus genomes identified from a sewage oxidation pond sample

Accession no.	Isolate name	Genome length (nt)	GC content (%)	No. of reads	Coverage depth (×)	Top BLASTp hit of MCP^*a*^	
						Accession no.	% pairwise identity
MT309919	BS1_596	3,797	50.1	782	19	MK765641	42.50
MT309920	BS1_594	3,808	36	573	13	MT309929	50.50
MT309921	BS1_567	3,926	41.7	10,000	228	MH617746	59.10
MT309922	BS1_560	3,940	33.8	7,405	169	MT309982	53.40
MT309923	BS1_557	3,955	52.6	962	22	MH617134	60.60
MT309924	BS1_556	3,959	34.4	16,751	379	MT309925	67.10
MT309925	BS1_555	3,986	39.5	616	14	MT309934	67.50
MT309926	BS1_554	3,982	47	21,416	481	MH992209	55.60
MT309927	BS1_546	4,015	36.8	1,305	29	MH617584	76.70
MT309928	BS1_540	4,058	49.4	484	11	MT309925	64.30
MT309929	BS1_533	4,062	39.1	794	18	MH617567	58.00
MT309930	BS1_525	4,090	56.5	3,747	82	MH616782	59.60
MT309931	BS1_516	4,173	58.5	440	9	MH617074	69.80
MT309932	BS1_515	4,119	36.2	874	23	MH992221	57.90
MT309933	BS1_508	4,136	42.6	956	21	MT309925	60.70
MT309934	BS1_502	4,214	43.9	710	15	MT309925	67.70
MT309935	BS1_501	4,165	39.4	22,154	477	MK765646	52.70
MT309936	BS1_499	4,175	41.6	48,158	1,037	MK496825	84.30
MT309937	BS1_492	4,243	47.9	515	11	MT309925	63.70
MT309938	BS1_489	4,219	42	40,633	863	MT309937	54.70
MT309939	BS1_487	4,265	42.5	136,661	2,879	KP823396	83.40
MT309940	BS1_485	4,269	43.2	804	17	MK496822	76.30
MT309941	BS1_484	4,248	42.8	8,148	175	MT310016	88.40
MT309942	BS1_481	4,245	42	22,246	470	MH617700	63.40
MT309943	BS1_477	4,253	40.3	21,294	450	MH572427	85.70
MT309944	BS1_474	4,263	41.7	73,932	1,560	MH572292	87.20
MT309945	BS1_471	4,307	55.8	594	12	MH572285	63.30
MT309946	BS1_466	4,271	47.8	1,971	41	MH616977	65.70
MT309947	BS1_462	4,295	41.8	63,579	1,333	MT310014	90.80
MT309948	BS1_450	4,311	49.8	1,556	32	MH510271	84.20
MT309949	BS1_449	4,312	40.8	6,084	126	MH572283	81.80
MT309950	BS1_448	4,316	46.8	3,066	65	MH992170	79.10
MT309951	BS1_446	4,319	36.9	712	15	MH992221	51.80
MT309952	BS1_441	4,383	53	453	9	MH617103	62.40
MT309953	BS1_440	4,380	51.5	880	18	MH617138	66.50
MT309954	BS1_438	4,373	41.5	22,096	453	MT310014	85.50
MT309955	BS1_432	4,346	44.4	633	13	MN582062	70.20
MT309956	BS1_426	4,359	52.8	1,141	23	MT309988	79.90
MT309957	BS1_425	4,360	50.3	8,885	183	MH510271	78.20
MT309958	BS1_423	4,362	48.1	1,299	27	MT309957	74.70
MT309959	BS1_415	4,385	46.9	796	16	MT309983	59.90
MT309960	BS1_413	4,386	42.2	3,251	67	MK496825	74.80
MT309961	BS1_412	4,387	51.4	1,428	29	MH622930	89.80
MT309962	BS1_410	4,394	41.1	2,703	55	MH617735	72.80
MT309963	BS1_408	4,415	47	435	9	MT309928	44.40
MT309964	BS1_401	4,412	47.9	6,700	137	MT309999	75.90
MT309965	BS1_400	4,437	48.1	43,718	884	MT309968	87.40
MT309966	BS1_399	4,414	47.7	600	12	MT309984	67.50
MT309967	BS1_398	4,414	41.2	692	14	MK765582	58.30
MT309968	BS1_397	4,415	47.9	2,023	41	MT309965	87.40
MT309969	BS1_387	4,439	37.7	10,731	219	MH572285	48.48
MT309970	BS1_386	4,442	47.8	487	10	MH617685	77.60
MT309971	BS1_385	4,442	46.1	1,191	24	MT309978	62.80
MT309972	BS1_384	4,443	57.5	1,379	28	MK249220	59.60
MT309973	BS1_380	4,451	37.5	1,103	22	MT309992	72.70
MT309974	BS1_378	4,453	49	1,020	21	MK249212	72.10
MT309975	BS1_377	4,453	47.2	1,435	29	MH617260	67.20
MT309976	BS1_373	4,462	45.2	1,485	30	MK249225	68.30
MT309977	BS1_372	4,463	38.8	1,090	22	MT309935	47.50
MT309978	BS1_370	4,516	43	702	14	MT309984	73.50
MT309979	BS1_368	4,474	38.5	9,022	181	MH992203	66.10
MT309980	BS1_367	4,481	49	42,406	853	MT310017	89.50
MT309981	BS1_364	4,487	59.7	2,315	46	MH552548	62.40
MT309982	BS1_361	4,494	35.1	92,459	1852	MT309922	53.40
MT309983	BS1_360	4,497	44.3	3,457	69	MK249212	66.90
MT309984	BS1_352	4,516	43.6	731	15	MT309978	73.50
MT309985	BS1_351	4,516	47	2,031	41	MK249219	82.30
MT309986	BS1_350	4,519	48.9	4,940	98	MT310017	81.20
MT309987	BS1_348	4,524	58.1	26,775	532	MK249179	80.60
MT309988	BS1_347	4,555	53.1	854	17	MH616807	76.90
MT309989	BS1_340	4,539	47.7	872	17	MT310007	65.50
MT309990	BS1_339	4,591	58	1,020	20	MH617422	70.00
MT309991	BS1_336	4,546	46.9	1,163	23	MT309961	62.60
MT309992	BS1_330	4,552	36.9	16,467	325	MT309973	72.70
MT309993	BS1_328	4,561	49.5	1,440	28	KP087956	49.40
MT309994	BS1_322	4,632	52.6	9,817	190	MK249189	70.80
MT309995	BS1_320	4,578	53.2	1,410	28	MK249190	68.00
MT309996	BS1_313	4,587	46	2,754	54	MH617260	67.80
MT309997	BS1_312	4,589	47.3	906	18	MH992193	61.70
MT309998	BS1_311	4,591	48.6	5,310	104	MH617374	72.40
MT309999	BS1_308	4,596	47.5	1,450	28	MT309963	75.90
MT310000	BS1_305	4,605	48.9	1,519	30	MT309980	82.30
MT310001	BS1_300	4,620	50.9	1,239	24	MK249178	79.40
MT310002	BS1_295	4,635	49.1	702	14	MK249163	78.10
MT310003	BS1_294	4,636	46.9	2,299	45	MH572492	62.30
MT310004	BS1_286	4,666	49.1	6,498	125	MT309966	60.80
MT310005	BS1_285	4,683	33.3	633	14	MH617644	36.90
MT310006	BS1_280	4,701	48.8	3,097	59	MH616638	85.50
MT310007	BS1_270	4,727	45.8	811	15	MT309989	65.00
MT310008	BS1_264	4,765	51.7	1,699	32	MH616735	71.64
MT310009	BS1_260	4,789	50.7	1,003	19	MH572441	66.70
MT310010	BS1_250	4,918	43.1	2,415	44	MH992183	62.60
MT310011	BS1_235	5,009	33.8	855	17	MH617129	54.60
MT310012	BS1_228	5,043	46.1	777	14	KT264751	98.40
MT310013	BS1_206	4,600	46.7	67,641	1,324	MH616922	78.10
MT310014	BS1_34	4,361	43.1	17,779	366	MT309947	90.80
MT310015	BS1_33	4,363	42.1	38,905	803	MH572427	78.90
MT310016	BS1_31	4,230	42.6	8,662	185	MT309941	88.40
MT310017	BS1_11	4,520	48.7	41,024	819	MT309980	89.50

a MCP, major capsid protein.

This study adds to the growing data on microviruses from various ecosystems and certainly shows that this group of viruses is diverse with varied genome organization.

### Data availability.

The sequences of microviruses in this study have been deposited in the NCBI SRA under project SRR11451582 and GenBank accession numbers MT309919 to MT310017.
